# Comprehensive Management of Vasculitis and Suspected Polyarteritis Nodosa in an Older Patient

**DOI:** 10.7759/cureus.36307

**Published:** 2023-03-17

**Authors:** Ryuichi Ohta, Chiaki Sano

**Affiliations:** 1 Community Care, Unnan City Hospital, Unnan, JPN; 2 Community Medicine Management, Shimane University Faculty of Medicine, Izumo, JPN

**Keywords:** testicular pain, rural, general medicine, japan, older, polyarteritis nodosa

## Abstract

Polyarteritis nodosa (PAN) is a rare autoimmune disease that affects medium-sized arteries and causes inflammation and damage to the blood vessel walls. Testicular pain is an uncommon symptom of PAN but can occur in rare cases. This specific symptom may be useful in diagnosing older patients with limited tissue access because of their vulnerability and high risk for biopsy complications. We report the case of a 78-year-old male patient with progressive fatigue and walking difficulty. After ruling out various forms of vasculitis and malignancy, we diagnosed the patient with PAN and intensively treated him with rituximab, which successfully cured his symptoms. This case report highlights the importance of intensively ruling out possible diagnoses mimicking vasculitis and treating vasculitis with a tentative diagnosis of PAN in older patients in rural hospitals. The progressive clinical course of vasculitis may devastate older patients’ activities of daily living (ADLs). PAN may particularly affect older patients with possible hepatitis B infections. Thus, shared decision-making and prompt intensive treatment should be considered.

## Introduction

Polyarteritis nodosa (PAN) is a rare autoimmune disease that affects medium-sized arteries and causes inflammation and damage to the blood vessel walls [1]. The disease is more common in older adults, and its diagnosis can be challenging because of its nonspecific symptoms [2]. PAN presents with various symptoms; precise history-taking and physical examination are essential [3]. Precise medical history can identify risk factors for PAN, such as a history of hepatitis B or C infection [1]. However, older patients with PAN often show vague symptoms, such as fever, weight loss, fatigue, and muscle or joint pain, similar to other autoimmune diseases [4,5]. Physical examination of skin rashes, ulcers, or nodules may indicate vasculitis. Various imaging tests, such as computed tomography (CT) and magnetic resonance imaging, can detect arterial abnormalities [1]. A biopsy of the affected tissue may confirm the diagnosis of PAN. However, detecting arterial abnormalities can be challenging because of the rapid progression of vasculitis in older patients [3].

Testicular pain is an uncommon symptom of PAN but can occur in rare cases [3]. This specific symptom may be useful in diagnosing older patients with limited tissue access because of their vulnerability and high risk for biopsy complications [6]. A tentative diagnosis of PAN is essential for effective treatment because vasculitis can progress rapidly, impinge the quality of life, and increase mortality [2]. Here, we report the case of an older male patient with progressive fatigue and difficulty walking. After ruling out various forms of vasculitis and malignancy, we diagnosed the patient with PAN and intensively treated him with rituximab and prednisolone, which successfully cured his symptoms. This case report discusses the limitations of PAN diagnosis and treatment methods in a rural context with limited resources.

## Case presentation

A 78-year-old male visited a rural community hospital with chief complaints of fatigue and low-grade fever for three months. Three months before admission, the fatigue gradually worsened while working as a restaurant chef. Two months before admission, the patient developed bilateral extremity edema. One month before admission, his temperature increased to 37°C, for which he was treated in a primary care clinic with acetaminophen. After treatment, his fatigue and low-grade fever persisted, and he could not work. Therefore, he visited our hospital. Two weeks prior, he had experienced persistent testicular and back pain. He reported no weight loss, chest pain, cough, abdominal pain, or diarrhea. The patient had a medical history of hypertension and diabetes, for which he was treated with amlodipine (5 mg daily) and metformin (750 mg daily).

His vital signs at the visit were as follows: blood pressure, 156/96 mmHg; pulse rate, 63 beats/minute; body temperature, 37.8°C; respiratory rate, 18 breaths/minute; and oxygen saturation, 90% on room air. The patient was alert to time, place, and person. Physical examination revealed distension of the jugular veins, distant sounds of the heart without murmur, abdominal distension without tenderness, bilateral livedo reticularis on the front of the thighs, and disappearance of the bilateral Achilles and knee-tendon reflexes. No other neurological abnormalities were observed. No abnormalities were observed on the chest, abdomen, or skin eruptions. Physical examination of the right testis revealed heating and tenderness. Laboratory tests showed an increased white blood cell count (neutrophils, 89%), high C-reactive protein level, and elevated erythrocyte sedimentation rate without kidney and liver abnormalities. Tests revealed no active hepatitis B virus (HBV) infection (Table 1).

**Table 1:** Initial laboratory data of the patient eGFR, estimated glomerular filtration rate; CK, creatine kinase; CRP, C-reactive protein; Ig, immunoglobulin; HBV: hepatitis B virus, HCV, hepatitis C virus; SARS-CoV-2, severe acute respiratory syndrome coronavirus 2; HBs, hepatitis B surface antigen; HBc, hepatitis B core antigen; C3, complement component 3; C4, complement component 4; MPO-ANCA, myeloperoxidase antineutrophil cytoplasmic antibody; PR3-ANCA, proteinase 3 antineutrophil cytoplasmic antibody; SS, Sjögren’s syndrome; CCP, cyclic citrullinated peptide; S/CO, sample/cutoff

Parameter	Level	Reference
White blood cells	11.2	3.5-9.1 × 10^3^/μL
Neutrophils	69.9	44%-72%
Lymphocytes	14.6	18%-59%
Monocytes	14	0%-12%
Eosinophils	0.8	0%-10%
Basophils	0.7	0-3%
Red blood cells	3.27	3.76-5.50 × 10^6^/μL
Hemoglobin	9.8	11.3-15.2 g/dL
Hematocrit	30.5	33.4%-44.9%
Mean corpuscular volume	93.2	79-100 fL
Platelets	24.4	13-36.9 × 10^4^/μL
Erythrocyte sedimentation rate	78	2-10 mm/hour
Total protein	7.2	6.5-8.3 g/dL
Albumin	3.4	3.8-5.3 g/dL
Total bilirubin	1.1	0.2-1.2 mg/dL
Aspartate aminotransferase	14	8-38 IU/L
Alanine aminotransferase	11	4-43 IU/L
Alkaline phosphatase	70	106-322 U/L
Lactate dehydrogenase	182	121-245 U/L
Blood urea nitrogen	16.4	8-20 mg/dL
Creatinine	1	0.40-1.10 mg/dL
eGFR	55.3	>60 mL/minute/L
Serum Na	141	135-150 mEq/L
Serum K	4.2	3.5-5.3 mEq/L
Serum Cl	102	98-110 mEq/L
Serum Ca	10	3.5-5.3 mg/dL
Serum P	3.4	0.2-1.2 mg/dL
Serum Mg	1.8	1.8-2.3 mg/dL
Ferritin	44.5	14.4-303.7 ng/mL
CK	46	56-244 U/L
CRP	10.16	<0.30 mg/dL
IgG	1,366	870-1,700 mg/dL
IgM	135	35-220 mg/dL
IgA	775	110-410 mg/dL
IgE	281	<173 mg/dL
HBs antigen	0	IU/mL
HBc antibody	3.83	S/CO
HBV DNA	Negative	Negative
HCV antibody	0	S/CO
Syphilis treponema antibody	0	S/CO
SARS-CoV-2 antigen	Negative	Negative
Antinuclear antibody	40	<40
Homogeneous	40	<40
Speckled	40	<40
C3	130	86-164 mg/dL
C4	24	17-45 mg/dL
MPO-ANCA	<1	<3.5 U/mL
PR3-ANCA	<1	<3.5 U/mL
Anti-SS-A/Ro antibody	<1	<10 U/mL
Anti-CCP antibody	<0.6	<5 U/mL
Urine test		
Leukocyte	Negative	Negative
Nitrite	Negative	Negative
Protein	(1+)	Negative
Glucose	(-)	Negative
Urobilinogen	Normal	Normal
Bilirubin	Negative	Negative
Ketone	Negative	Negative
Blood	Negative	Negative
pH	6.5	
Specific gravity	1.013	

Chest radiography revealed bilateral pleural effusion and cardiac enlargement.

**Figure 1:**
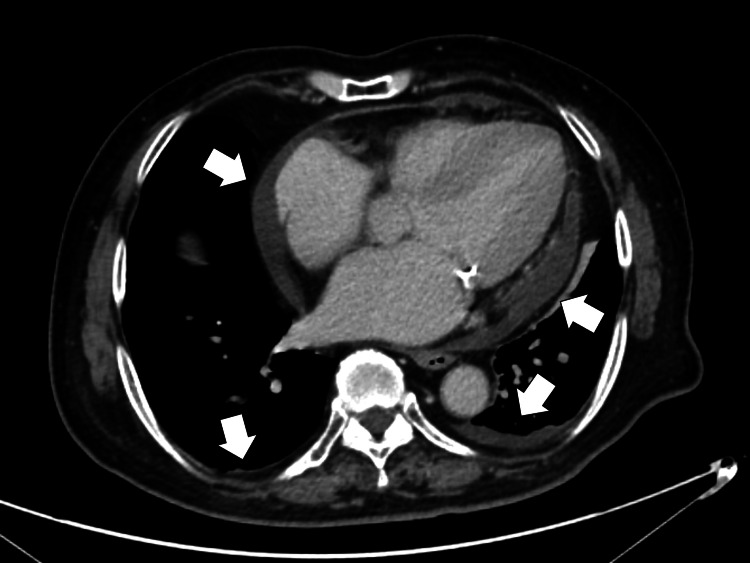
Chest computed tomography with contrast showing bilateral pleural and pericardial effusions (white arrows)

During admission, his fever persisted at around 37.5-38.7°C, and fatigue and appetite loss worsened. On the fifth day of admission, further investigation of the bilateral effusions and heart enlargement using chest CT with contrast showed bilateral pleural and pericardial effusions (Figure 1).

**Figure 2:**
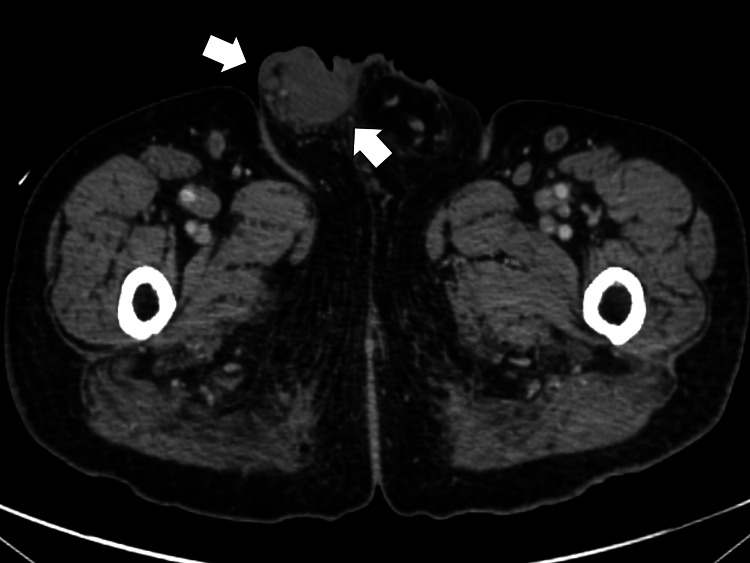
Pelvic computed tomography showing right testicular enlargement with edema (white arrows)

In addition, contrast-enhanced abdominal CT revealed edema, segmented superior and inferior mesenteric artery dilation, and right testicular enlargement with edema (Figure 2).

On the sixth day of admission, a skin biopsy of the lesions was performed to investigate further the livedo reticularis, which showed negative results for arteritis. On the same day, nerve conduction velocity tests were performed on the bilateral sural, tibial, and fibular nerves to investigate peripheral neuropathy, revealing bilateral axonopathies of the tibial and fibular nerves. Investigating bacteremia using four sets of blood culture tests and miliary and latent tuberculosis with interferon-gamma release assays performed on the initial admission day yielded negative results. On the 10th day of admission, random biopsies were performed to investigate intravascular lymphoma (IVL); however, the results were negative for malignancy.

Based on the clinical findings, PAN was suspected. After discussing the condition with the patient and his family, intensive treatment was provided for the suspected PAN. The patient was treated with prednisolone (1 mg/day), followed by rituximab (500 mg/week) four times. After treatment, the patient’s symptoms gradually disappeared within a week. Due to progressive frailty, the patient was rehabilitated in the rehabilitation unit with tapering of prednisolone at a rate of 10 mg/two weeks. As his activities of daily living (ADLs) improved, he was discharged on the 42nd day with prednisolone (20 mg) and followed up at the outpatient department.

## Discussion

This case report highlights the importance of intensively ruling out possible diagnoses mimicking vasculitis and treating vasculitis with a tentative diagnosis of PAN in older patients in rural hospitals. The progressive clinical course of vasculitis can devastate ADLs in older patients. PAN may particularly affect older patients with possible hepatitis B infection. Thus, shared decision-making and prompt intensive treatment should be considered.

Therefore, it is essential to rule out any differential diagnoses. To effectively diagnose vasculitis, including PAN, various critical diseases, such as sepsis, infectious endocarditis (IE), and malignancies, including IVL, must be ruled out [7,8]. Vasculitis occurs mainly in older patients, and intensive investigations are limited because of frailty and self-agism [9,10]. The minimal requirements for ruling out differential diagnoses are blood cultures, systemic CT scans with contrast, and dermal biopsies [7,8]. Four blood cultures were used to rule out persistent bacteremia, including infectious endocarditis (IE) [11]. Systemic CT with contrast can be used to investigate malignancy’s invasive pathogens focus on inflammation, including abscess formation and lymph node swelling [7,8]. In addition, random skin biopsies can detect IVL [7,8]. These investigations may not rule out all possibilities of occult bacteremia and malignancy, but these critical diseases may be tentatively ruled out. Therefore, clinicians can progress to the next step in managing vasculitis through discussions with patients and their families.

Occult HBV infection can trigger PAN. The prevalence of HBV infection has decreased due to advances in new treatments [12]. Simultaneously, the incidence of HBV-induced PAN has decreased, particularly in developed countries [13]. However, PAN is prevalent even in the absence of overt HBV infection [13]. In contrast, occult HBV infection occurs in older individuals, and immunosuppressive treatments may induce de novo HBV hepatitis [14]. HBV infects various immunological cells, such as lymphocytes, and induces immunogenicity [15]. Occult HBV infection can trigger PAN. In this case, the patient was negative for hepatitis B surface antigen (HBsAg) but positive for HBc antibodies, indicating the possibility of an occult HBV infection. Investigation for occult HBV infection is mandatory in patients with suspected vasculitis, including those with PAN. However, the treatment of occult HBV infection is controversial because of the side effects of medications for HBV infections [15]. Our patient tested negative for HBV DNA; therefore, he was not treated with HBV antiviral drugs. The presence of signs of occult HBV infection, such as positivity for HBs and HBc antibodies, can support a diagnosis of PAN and requires meticulous follow-up to prevent de novo HBV hepatitis.

Shared decision-making and prompt intensive treatment are critical for caring for older patients with PAN. PAN requires intensive treatment with large amounts of prednisolone and immunosuppressants, such as cyclophosphamide and rituximab [2]. A tentative diagnosis may be inevitable in older patients with frailty and symptoms of vasculitides, such as progressive paralysis, cardiovascular inflammation, cardiac tamponade, and gastrointestinal bleeding [12]. However, these treatments are invasive and may lead to complications. Shared decision-making is thus mandatory for managing older patients [16]. Although the pathophysiology of vasculitis is complicated, physicians must explain the effectiveness and complications of treatment to their patients and decide on management plans. In the present case, the patient and his family wanted to undergo invasive treatment, which eventually led to symptom mitigation.

## Conclusions

This case report highlights the importance of intensively ruling out possible diagnoses mimicking vasculitis and treating vasculitis with a tentative diagnosis of PAN in older patients in rural hospitals. The progressive clinical course of vasculitis can devastate ADLs in older patients. Thus, shared decision-making and prompt intensive treatment should be considered.
